# A Green Solvent Induced DNA Package

**DOI:** 10.1038/srep09137

**Published:** 2015-03-16

**Authors:** Sagar Satpathi, Abhigyan Sengupta, V. M. Hridya, Krishna Gavvala, Raj Kumar Koninti, Bibhisan Roy, Partha Hazra

**Affiliations:** 1 Department of Chemistry, Indian Institute of Science Education and Research (IISER)-Pune, Pune (411008), Maharashtra, India

## Abstract

Mechanistic details of DNA compaction is essential blue print for gene regulation in living organisms. Many *in vitro* studies have been implemented using several compaction agents. However, these compacting agents may have some kinds of cytotoxic effects to the cells. To minimize this aspect, several research works had been performed, but people have never focused green solvent, i.e. room temperature ionic liquid as DNA compaction agent. To the best of our knowledge, this is the first ever report where we have shown that guanidinium tris(pentafluoroethyl)trifluorophosphate (Gua-IL) acts as a DNA compacting agent. The compaction ability of Gua-IL has been verified by different spectroscopic techniques, like steady state emission, circular dichroism, dynamic light scattering and UV melting. Notably, we have extensively probed this compaction by Gua-IL through field emission scanning electron microscopy (FE-SEM) and fluorescence microscopy images. We also have discussed the plausible compaction mechanism process of DNA by Gua-IL. Our results suggest that Gua-IL forms a micellar kind of self aggregation above a certain concentration (≥1 mM), which instigates this compaction process. This study divulges the specific details of DNA compaction mechanism by a new class of compaction agent, which is highly biodegradable and eco friendly in nature.

For safe storage of genetic information, compaction of DNA is appearing as a budding topic of interest. A normal living cell contains a huge number of DNA which in average consist around six billion DNA base pairs. Considering each base pair length ~0.34 nm, six billion DNA base pairs comprise a total distance of 2 meters. Including the total number of cells in a human body (nearly 50 trillion), an unbelievable length of 100 trillion meters of DNA per human appears in picture. Miraculously, nature manages this length of DNA inside a nucleus by the formation of a complex named as chromatin^1^. Not only a natural compaction but an artificial DNA compaction is also an effective way to store and carry genetic information^2^. Compacted DNA also found to be resistant/stable towards any external shocks like UV radiation^3^. Moreover, mechanically compacted DNA has recently been used as nanostructure template^4^ and also applied as protection against chemical, biochemical and mechanical stresses^5^. Learning from the nature, researchers have started applying the compaction tricks using polyamines^6^,^7^, surfactants^8^,^9^, liposomes^10^, nanoparticles^11^,^12^, polymer^13^, osmoticants like polyethylene glycol (PEG)^14^, dendrimers^15^,^16^, multivalent ions^17^, metal complex^18^, cyclodextrin^19^, peptides^20^ and proteins^21^ for compaction and storage of DNA for prolonged duration. Among these compacting agents, most important naturally occurring DNA compaction agents are proteins and polyamines like of spermine and spermidine etc^20^,^21^. Under physiological pH (~7.4), polyamines generally exists in positively charged form, which participates in strong electrostatic interaction with negatively charged phosphate backbone of DNA, and thereby results compaction and enhances the thermal stability of DNA^3^,^22^. Thus different types of linear and branched polyamines have been identified in many hyperthermophilic archaea^23^. Not only polyamines but several multivalent cations can also lead to DNA compaction by decreasing intra-strand DNA repulsion^24^. Surfactants with positively charged head group can act as multivalent cations above critical micellar concentration (CMC), which can neutralize the negatively charged DNA surface, and hence facilitates the compaction process^9^. Moreover, hydrophobicity of surfactants tails also play a crucial role for DNA compaction^8^. Recently Ganguli *et al.* have used positively charged nanoparticles to condense DNA strands^11^. Instead of forming a completely compacted DNA, it generates several compacted small loops of DNA around each nanoparticles^11^. In contrary to Ganguli *et al.*, Rudiuk *et al.* reported that negatively charged silica nanoparticle improves the surfactant effectivity for DNA compaction^25^. Moreover Zinchenko *et al.* mentioned that negatively charged silica (SiO_2_) nanoparticle can lead to DNA compaction by causing depletion in DNA coil structure due to the excluded volume of nanoparticles^12^. Not only ions, osmoticants, like organic solvents, polyethylene glycol (PEG) can also induce DNA compaction by lowering the dielectric permittivity of solution^26^. Yoshikawa *et al.* studied the DNA compaction by polyethylene glycol with pendant amino groups (PEG-A). They have observed that DNA is collapsed by PEG-A similar to neutral PEG, but it happens at lower concentration^14^. Dendrimers are also considered as one of the most efficient DNA compacting agent. Its compaction ability depends upon size and charge distribution. Generally, dendrimers used at higher dendrimers-to-DNA charge ratio (5–10) account for their brilliant DNA compacting ability^15^. But highly charged dendrimers are very toxic to living cells. Thus, several research groups studied this mode of compaction process using modified dendrimer surface by acetylation, PEGylation^27^ etc. Besides these substances, researchers have also studied the impact of chirality on DNA compaction by using chiral polycations and chiral polypeptide^7^,^28^. Interesting reports are also available about reversibly controlled compaction/decompaction process of DNA^29^.

Since the discovery of ionic liquid (IL) in 1965^30^, it has been attracted significant attention among researchers due to its unique properties like low vapor pressure, inflammability, very high thermal, chemical stability and excellent conductivity^31^,^32^. Thus IL has been exploited in various kind of research field like separation, synthesis, electrochemistry, catalysis etc^31^,^33^. Considering its low cytotoxicity and high biodegradability, many groups have studied the interaction of room temperature ionic liquid (RTIL) with biomacromolecules like protein, DNA etc^34^,^35^. Nevertheless, literature of DNA-IL interaction is somewhat controversial due to several contradictory explanations provided by different reports. First, Cheng *et al.*^36^ suggested that cationic part of [Bmim][PF_6_] can intercalate inside the DNA base pairs, whereas later Ding *et al.*^37^ indicated an aggregation induced DNA perturbation by [Bmim][Cl] IL. Very recently in 2012, Chandran *et al.*^38^ proposed a groove binding mechanism of RTIL in DNA, where they have shown that [Bmim] cation interacts with minor groove of DNA through various non covalent interactions. Thus, it is clear that mode of DNA-IL interaction is still ambiguous and this may be due to the inertness of IL towards any thermal and optical signals.

Generally, a substance, which can increase the intrastrand/interstrand attraction between DNA molecules, is treated as DNA compacting agent. ILs contain cationic part which can compensate DNA backbone charges through electrostatic interaction, and may lead to DNA compaction through the formation self aggregated polyvalent (containing multiple positive and negative charges) structure. Notably, micellar like aggregated structure of ILs are familiar in literature^39^, and can behave as a multivalent ion to facilitate DNA compaction process. Although IL pursues all potential character of a DNA compaction agent, it has not been considered as a potential DNA compacting agent. In this study, we have showed the DNA compaction ability of a room temperature ionic liquid (RTIL), namely, guanidinium tris(pentafluoroethyl) trifluorophosphate (Gua-IL) (Figure 1) using different spectroscopic techniques like UV-Vis spectroscopy, fluorescence, steady state anisotropy, circular dichroism (CD). We have also proposed the detail plausible mechanism of DNA compaction by Gua-IL using dynamic light scattering (DLS), scanning electron microscopy (SEM) and tunneling electron microscopy (TEM), confocal fluorescence microscopy along with above mentioned spectroscopic techniques. Unlike other compaction agents, we found IL stands as a better one due to its biodegradability and low concentration effectivity, which might be useful for extraction and storage of genetic blue prints for future.

## Results

### Steady state emission

**Figure 2:**
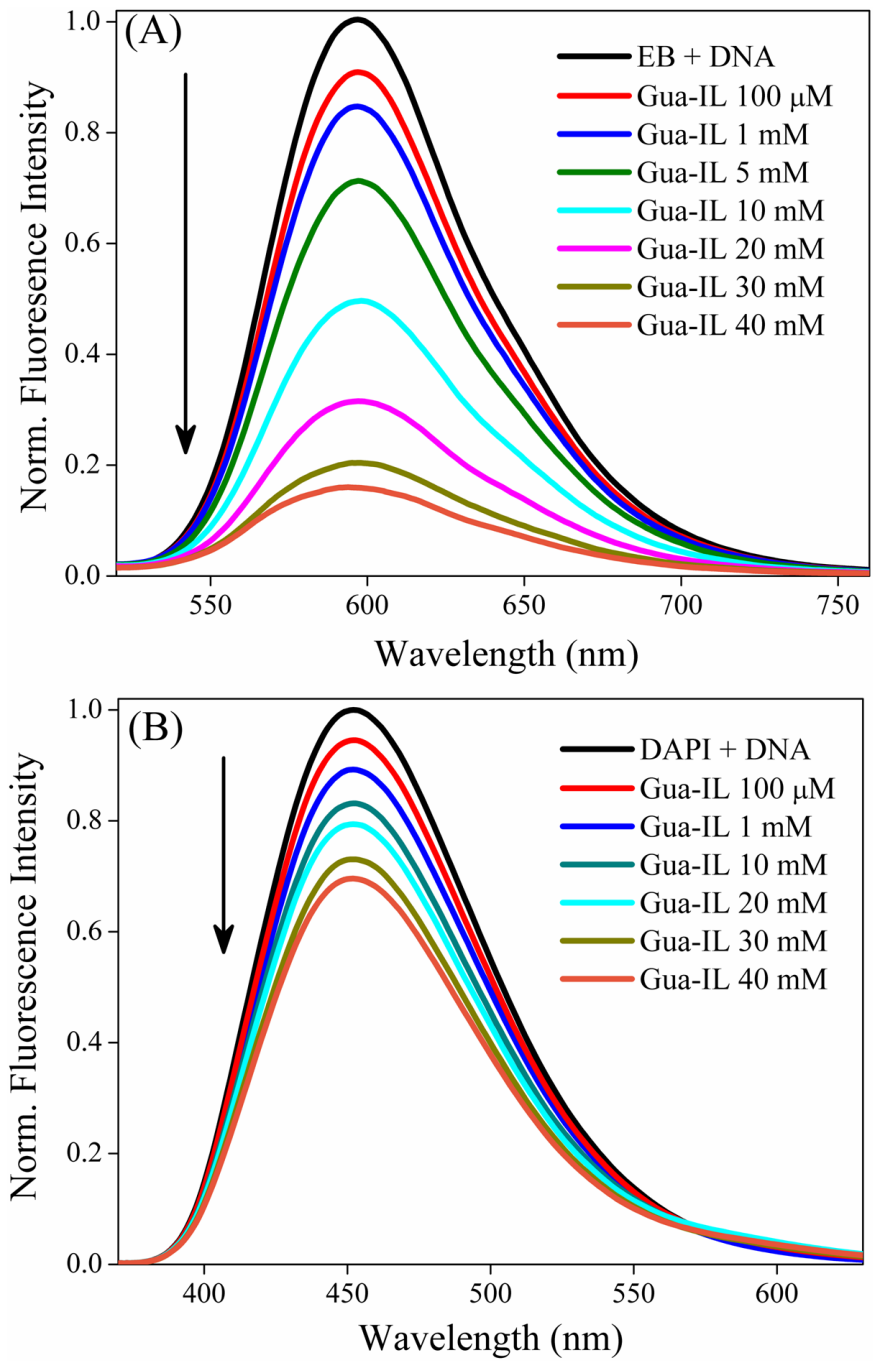
Emission profiles of dye displacement study. Fluorescence spectra of dye bound ct-DNA (~200 μM) ((A) EB (B) DAPI) with increasing concentration of Gua-IL.

From the last few decades steady state fluorescence spectroscopy has been emerged as a powerful technique for characterization of association properties between small molecules and nucleic acids. As both of guanidinium tris(pentafluoroethyl)trifluorophosphate (Gua-IL) and DNA do not exhibit any fluorescence, studies have been carried out using two different DNA binding probes; a well known intercalator i.e. ethidium bromide (EB) and a minor groove binder i.e. 4′,6-diamidino-2-phenylindole (DAPI). We have performed displacement assay experiments by these two DNA binding dyes for characterization of Gua-IL and ct-DNA interaction and the results are shown in Figure 2. Fluorescence intensity of DNA-bound EB is significantly higher than that of unbound EB (Figure S1A in SI) due to its insertion in more hydrophobic microenvironment (i.e. in between base pairs) and also for the less accessibility of intercalated EB by quenchers like H_2_O and/or dissolved O_2_^40^. Moreover, emission peak of EB (λ_ex_ at 478 nm) in Phosphate Buffer (PB) (pH = 7.4) appears at 610 nm, which shifts to 597 nm up to maximum addition of DNA (180 μM). When EB bound DNA solution is being titrated with Gua-IL, no significant change in emission maximum has been observed in micro-molar concentration range of Gua-IL, but in milli-molar concentration range (>1 mM) a strong quenching (~84% up to 40 mM of Gua-IL) in fluorescence intensity is observed (Figure 2A). To verify whether the changes in fluorescence spectral feature arise due to Gua-IL alone or not, we have done control experiment with Gua-IL alone (in absence of DNA), and we have noticed that no significant change is observed upon addition of Gua-IL to the solution containing EB (Figure S2A).

To get insight into the effect of Gua-IL over a DNA groove binder, we have carried out similar kind of steady state experiment with a well known minor groove binder, i.e. DAPI. DAPI exhibits emission maximum in bluish green region (at 483 nm) and a ~30 nm blue shift is observed along with increment in emission intensity with increasing ct-DNA concentration (Figure S1B). Figure 2B depicts the effect of Gua-IL on the fluorescence profiles of DNA-bound DAPI and the results exhibit a considerable decrease in emission intensity up to the maximum Gua-IL concentration. Interestingly, similar to EB, DAPI also shows a significant change in emission intensity only around milli-molar concentration range of Gua-IL. Note that the decrease in emission intensity at maximum addition of Gua-IL (40 mM) is not so effective like EB, inferring that Gua-IL cannot remove all the groove binder (DAPI), which indicates possible minor groove binding nature of Gua-IL. We have also performed control experiment with Gua-IL and DAPI and it indicates very negligible effect on emission maximum of DAPI (Figure S2B).

### Steady state anisotropy

**Figure 3:**
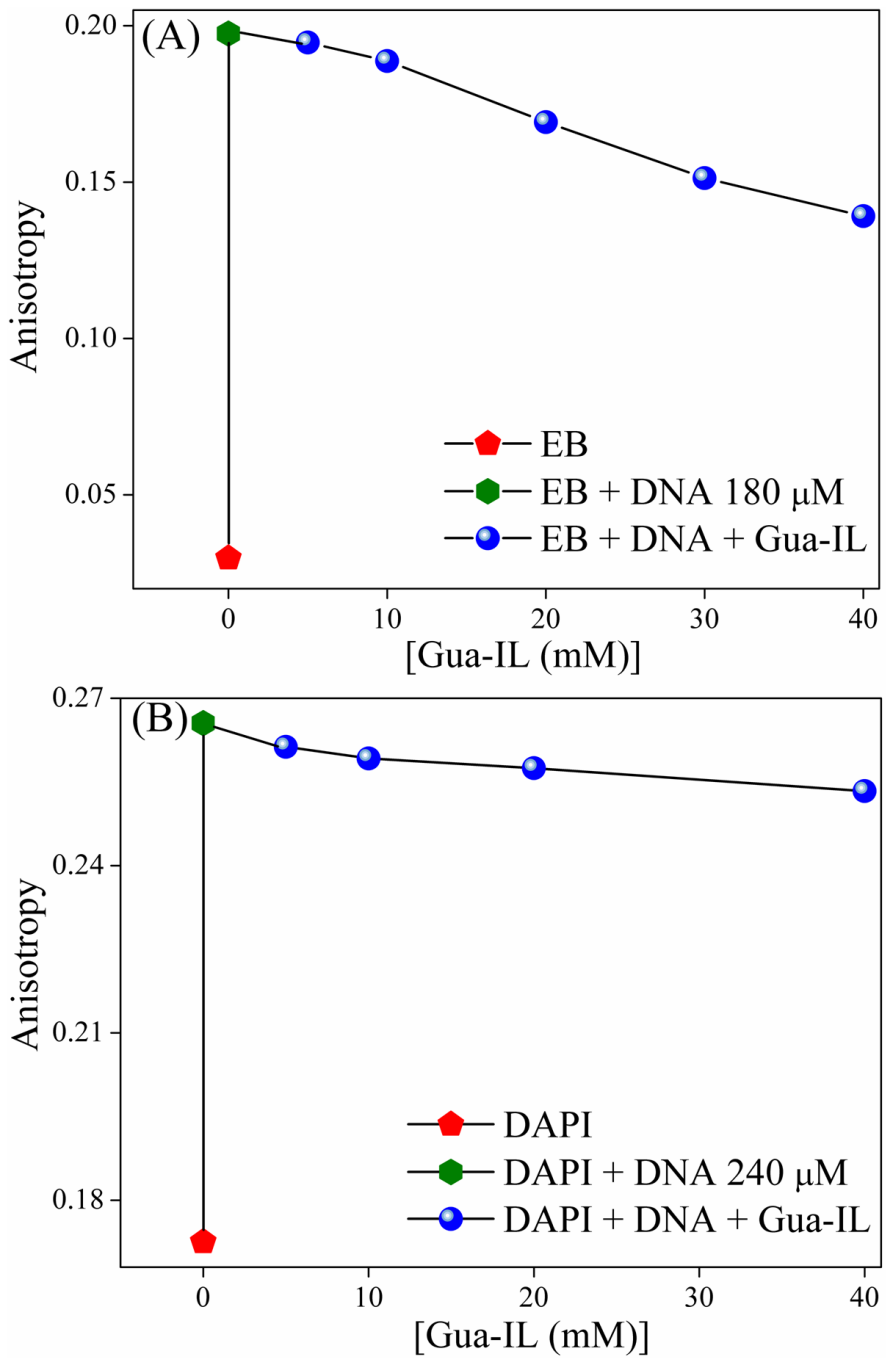
Steady state anisotropy of dye displacement studies. Change of steady state anisotropy of (A) EB (20 μM) (B) DAPI (20 μM) in presence of ct-DNA with increasing concentration of Gua-IL.

Fluorescence depolarization takes place due to rotational diffusion of the fluorophores in solvent medium^41^ and steady state fluorescence anisotropy is a measure of this depolarization or rotational diffusion of molecules^41^. A higher anisotropy value suggests slower rotational diffusion arising due to rigid environment around the molecule. With increasing addition of DNA in both EB and DAPI, we have observed enhancement (Figure 3A and 3B respectively) in anisotropy value, which corresponds to the interaction of dyes with respective sites of DNA. Interestingly, when Gua-IL is added to the solutions of EB-DNA and DAPI-DNA above milli-molar concentration (Figure 3A and 3B respectively), then anisotropy values for both the dyes decrease rapidly, although no such change in micro-molar concentration range is observed.

### Circular Dichroism (CD)

**Figure 4:**
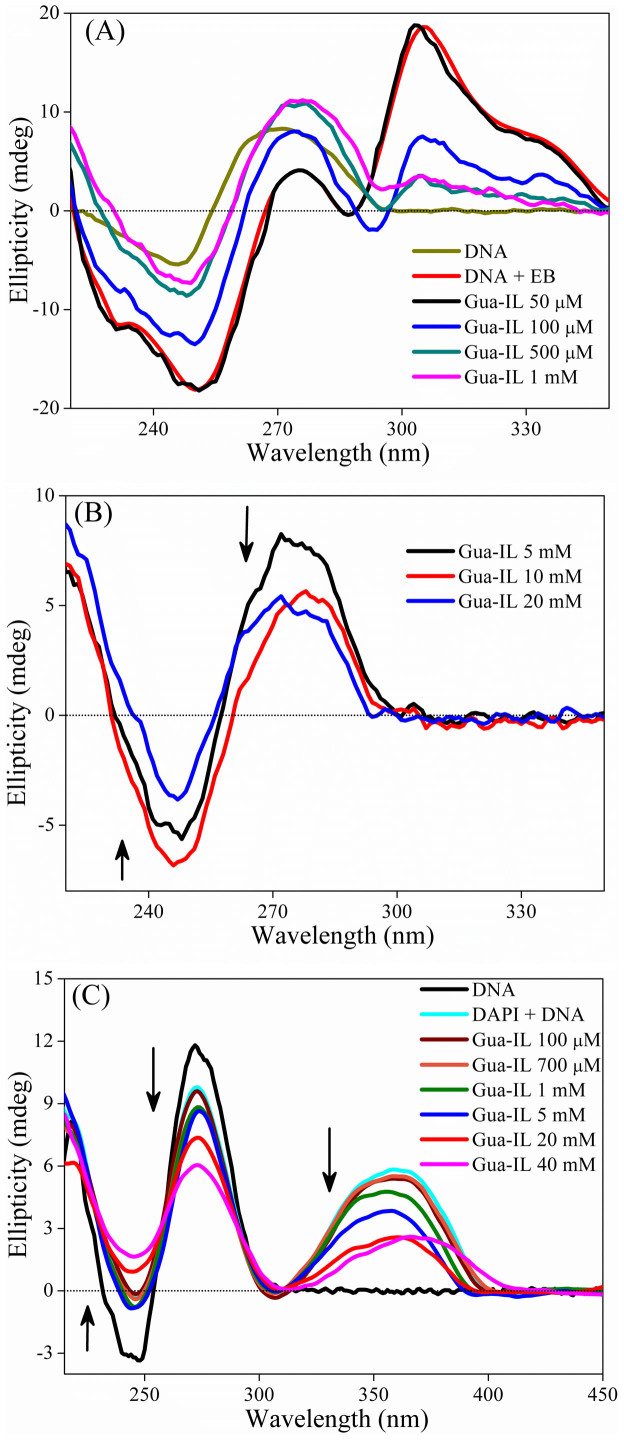
Circular dichorism study. Circular dichroism spectra of ct-DNA (~500 μM) with increasing concentration of dye and with micro-molar addition of Gua-IL in DNA-EB system (A), milli-molar addition of Gua-IL in DNA-EB system (B), DAPI and with addition of Gua-IL in DNA-DAPI system (C).

To gain insight about the DNA secondary structure perturbation by Gua-IL, we have next focused on circular dichroism measurements. ct-DNA exhibits two characteristics peaks (Figure S4 in SI); one positive band around 280 nm complemented to π-π base stacking and a negative band around 245 nm for hellicity^42^,^43^. While titrated with EB, DNA generates an induced CD signal near 303 nm (Figure S4) along with decreasing π-π stacking peak and increasing helicity peak, which is a well known feature for the intercalation of dye in DNA^44^,^45^. When Gua-IL is added to the DNA-EB system, no commendable variation in CD signal is noticed up to 100 μM of Gua-IL (Figure 4A). Afterward evident alteration is perceived in induced CD signal, helicity peak and base stacking peak. Notably, after 1 mM concentration of Gua-IL, induced CD signal completely vanishes which is suggestive of complete removal of intercalated EB from DNA (Figure 4B). Moreover, both the helicity and base stacking peak decreases from this concentration. In case of DNA-DAPI interaction, CD (Figure 4C) spectra show all sorts of characteristics features including an induced CD signal near 360 nm as per the reported literature^46^, which confirms the binding of DAPI to DNA. Addition of Gua-IL (Figure 4C) decreases the induced CD signal peak to a certain extent, inferring the replacement of DAPI molecules from the minor grove of ct-DNA. Interestingly, induced CD signal does not vanish completely as it is observed in case of DNA-EB system, indicating that some DAPI molecules still bind to DNA even at higher concentration of Gua-IL.

### Dynamic light scattering and zeta potential measurements

**Figure 5:**
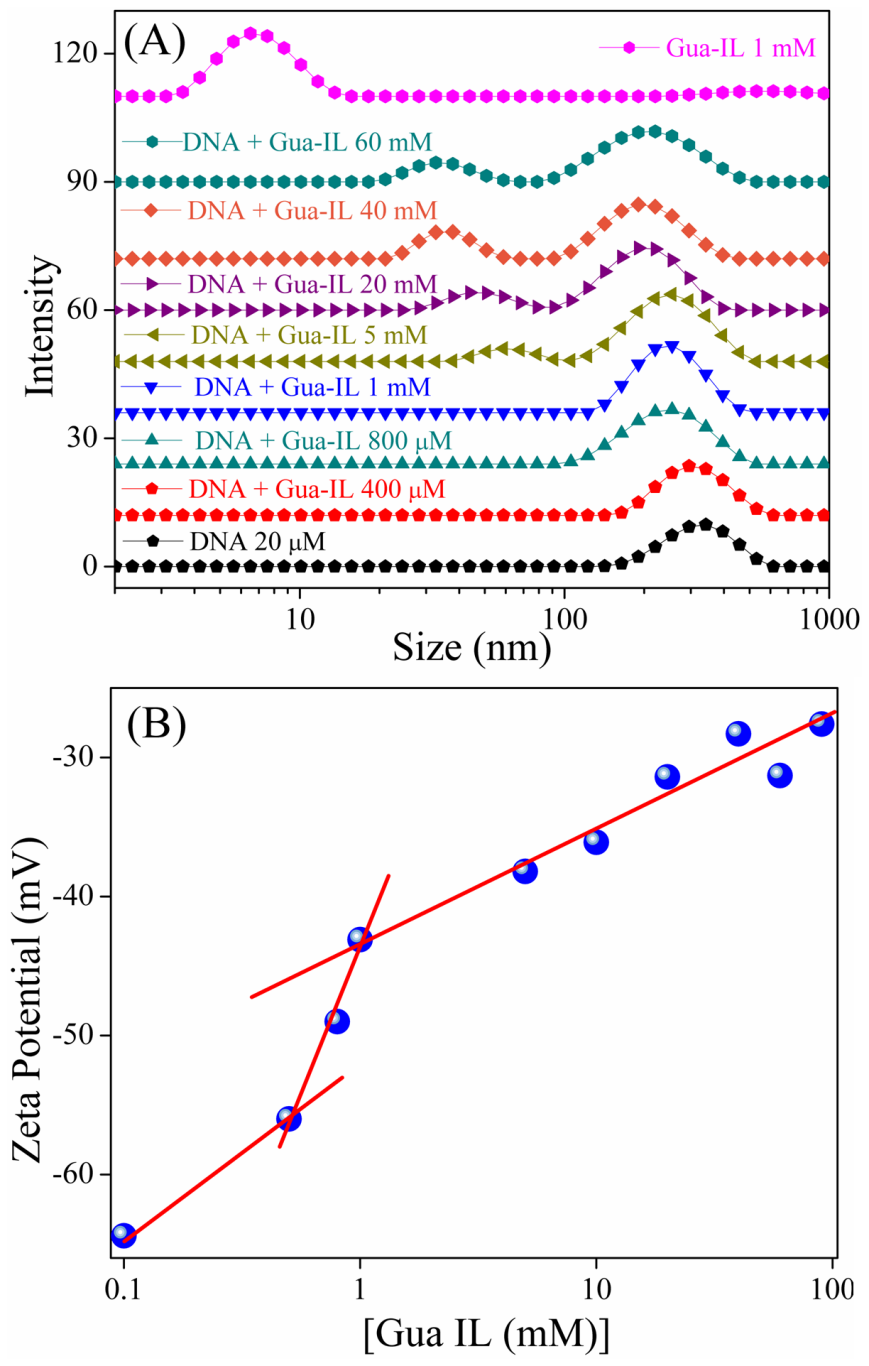
Dynamic light scattering and zeta potential study. (A) Intensity weighted distribution profile (measured by DLS) of ct-DNA (20 μM) with increasing concentration of Gua-IL. (B) Zeta potential plot as a function of Gua-IL concentration. X axes in both the figures are represented in logarithm scale.

For many years, dynamic light scattering is manifesting itself as a workhorse for measurement of size and shape of biological macromolecule. In order to clarify the nature of changes on DNA conformation by Gua-IL, we have carried out dynamic light scattering measurements. As the molecular weight of ct-DNA is high, we have performed experiments at low concentration of DNA (20 μM) in tris buffer (pH = 7.4) in order to avoid self interaction between the DNA molecules. The intensity weighted size distribution of DNA alone is found unimodal in nature and is presented on lower curve in Figure 5A. The mean hydrodynamic radius of DNA is about 350 nm, which is in agreement with the size of high molecular weight DNA^47^. When Gua-IL is added to DNA solution, the size distribution peak at 350 nm gradually shifted to lower hydrodynamic radius (~260 nm) and no new peak appears up to 1 mM concentration of Gua-IL. After ≥1 mM Gua-IL concentration, two distinct size distribution peaks emerge at 58 nm and 255 nm. In order to find out whether the above mentioned peaks appeared due to aggregation process of Gua-IL or not, we have carried out a DLS study in absence of DNA at different concentrations of Gua-IL (Figure S6). An intensity weighted distribution peak around 30–40 nm at higher concentration of Gua-IL is attributed to the aggregation of anionic part of Gua-IL. For further clarification about Gua-IL aggregation, we have performed a DLS study with guanidine hydrochloride (Gua HCl), which contains similar guanidinium cation like Gua-IL, but it contains chloride anion as counterpart. Guanidine hydrochloride does not show any intensity weighted distribution, which indicates the role of anionic part of Gua-IL for the aggregation process. In order to validate the DLS results, we have also executed zeta potential measurement experiment (Figure 5B) as a function Gua-IL concentration. Zeta potential is the electrostatic potential generated in an applied electric field due to attraction between charged species and oppositely charged electrode^48^. Zeta potential depends on location of the shear plane, which is the interface between stern and diffuse layers of the double layer^48^. Likewise, zeta potential of DNA depends on its conformation in accordance with the position of shear plane. Thus, zeta potential measurement is an excellent tool for determination of effective charge density, which in term is an essential parameter for DNA compaction. It is clear from Figure 5B that DNA shows a zeta potential value of −64 mV and it gradually increases with Gua-IL addition showing a visible inflection point near 1 mM of Gua-IL concentration.

### Thermal melting study

**Figure 6:**
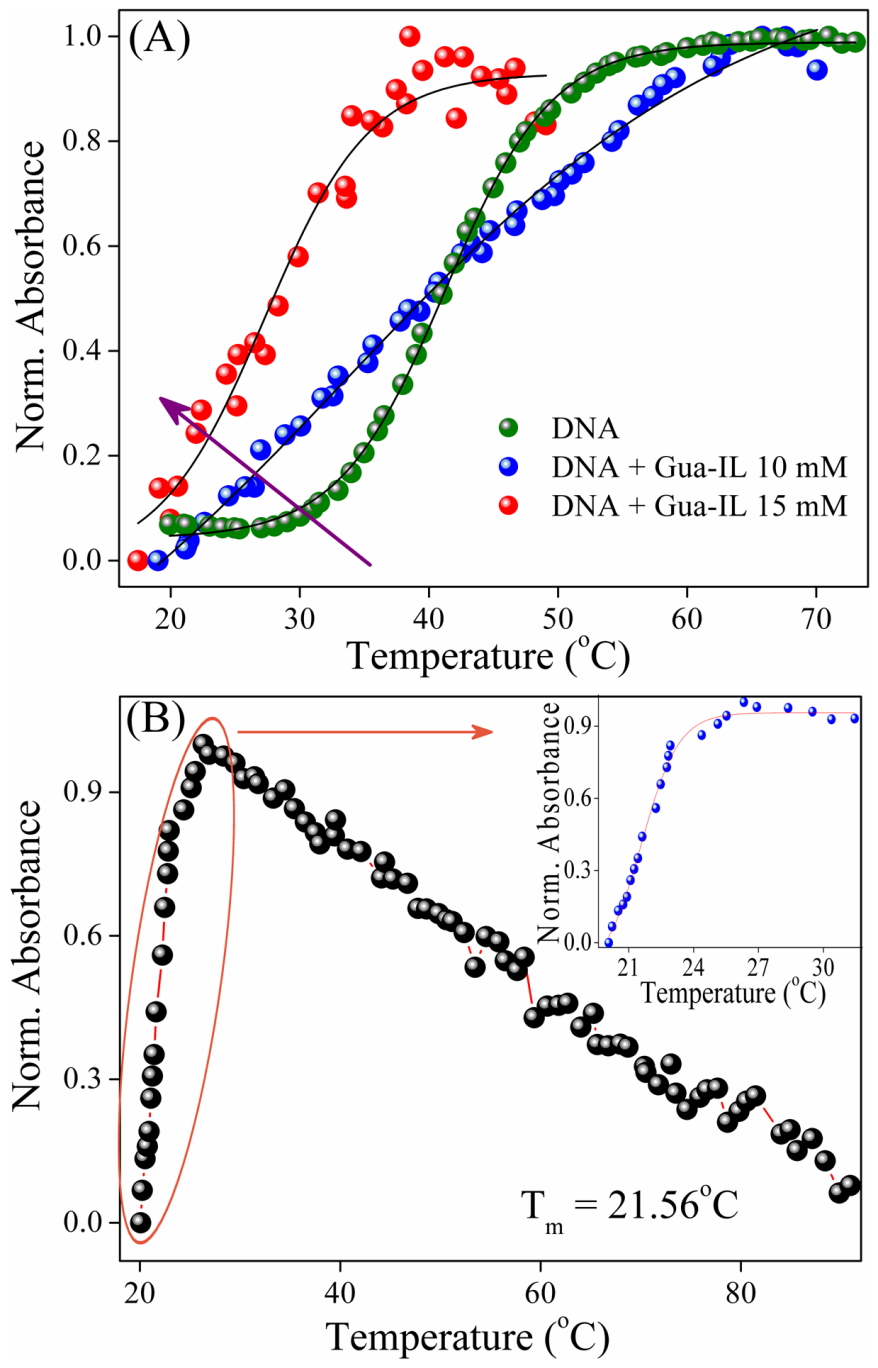
Thermal melting studies. UV melting studies of ct-DNA (~1 μM) in (A) different concentrations of Gua-IL (up to 15 mM) (B) in presence of 40 mM Gua-IL. Inset shows the fitting results.

UV melting experiment was performed with ct-DNA in absence and presence of Gua-IL in order to get insight into the structural stability of DNA by Gua-IL. Generally, with increase in temperature, double stranded DNA denatures to single stranded DNA with enhancement in absorbance at 260 nm due to unstacking of nucleobases and exhibits a transition point i.e. melting temperature. ct-DNA (1 μM) in tris-HCl buffer (pH = 7.4) shows a transition point at ~41°C and this melting point gradually shifted to lower temperature (~21°C) with increase in Gua-IL concentration (Figure 6).

### Microscopic view: field emission scanning electron microscopy (FE-SEM) and transmission electron microscopy (TEM) study

**Figure 7:**
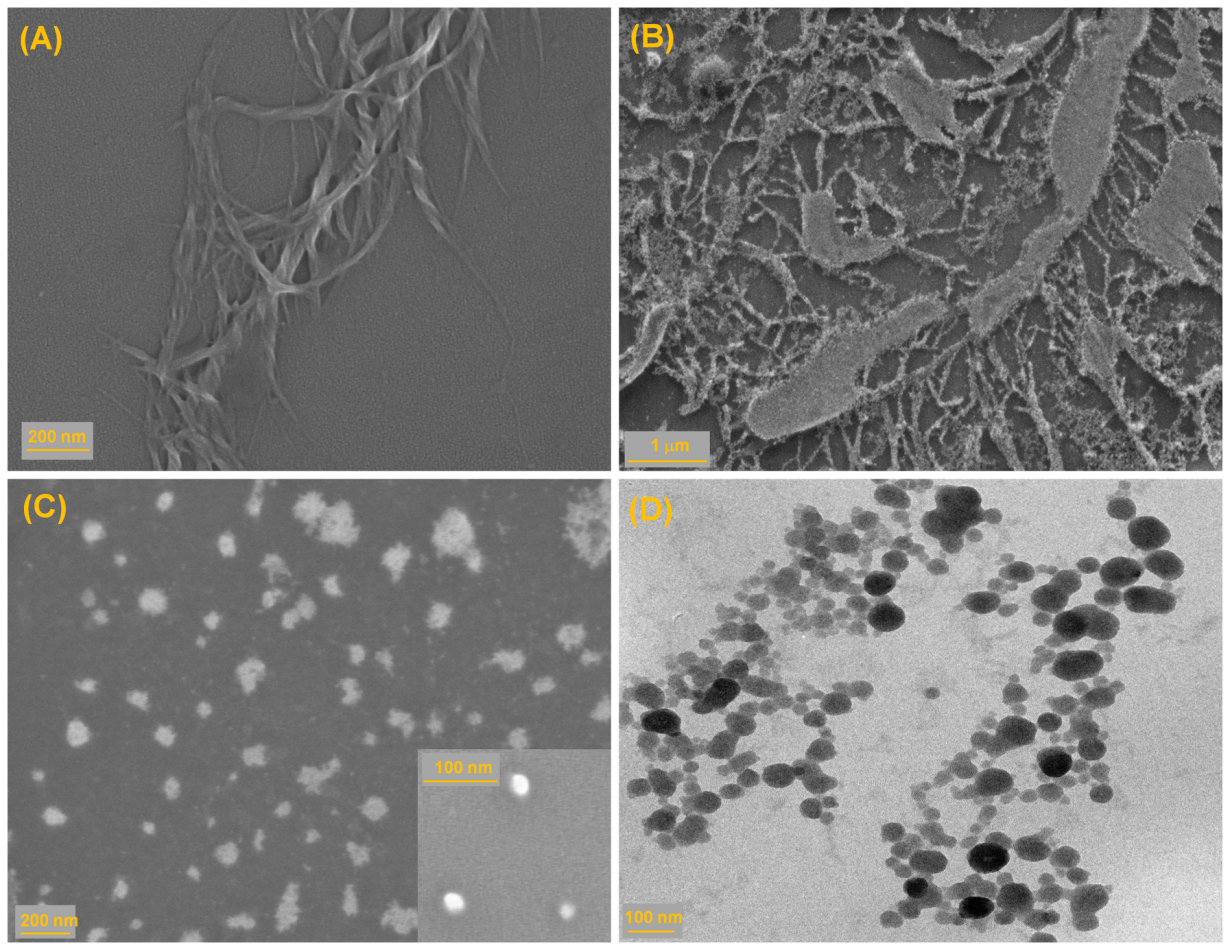
Microscopic images (i.e. FE-SEM and TEM). Microscopic FE-SEM images (A to C) in different molar ratio of [Gua-IL]/[ct-DNA]: (A) only ct-DNA (B) 20 (C) 40. (D) The TEM image of Gua-IL (5 mM).

Field emission scanning electron microscopy (FE-SEM) is used to probe the morphological alteration of DNA during compaction process induced by Gua-IL. All the FE-SEM samples are prepared in tris HCl (pH = 7.4) buffer to avoid any kind of artifact resulting from salt morphology. It is evident from Figure 7A that ct-DNA (in absence of Gua-IL) appears in super-coiled morphology, which is expected for a long DNA like calf thymus. When Gua-IL is gradually added, some morphological changes in the super-coiled DNA structure are observed depending on the molar ratio of IL to DNA, i.e., [Gua-IL]/[DNA]. At lower molar ratio 10 ([Gua-IL]/[DNA]), no specific difference in morphology is observed. A transition in morphology appears at molar ratio of 20 ([Gua-IL]/[DNA]), which is depicted in Figure 7B, and at high molar ratio of 40 ([Gua-IL]/[DNA]), a globular morphology appears in the context (Figure 7C). We have also probed the Gua-IL aggregation process in absence of DNA by FE-SEM and TEM studies (Figure 7D and Figure S7B). In both FE-SEM and TEM, we have found a distribution of sizes ranging from ~30 nm to ~200 nm. A closer look at the TEM images of Gua-IL (Figure 7D), it is evident that higher sized aggregated structures results from the smaller sized aggregates, and this is believed to appear due to drying effect of the sample during FE-SEM and TEM studies. Notably, size of these smaller aggregations is very much comparable with the size obtained from DLS study (i.e. ~30 nm).

### Fluorescence Microscopy Study

**Figure 8:**
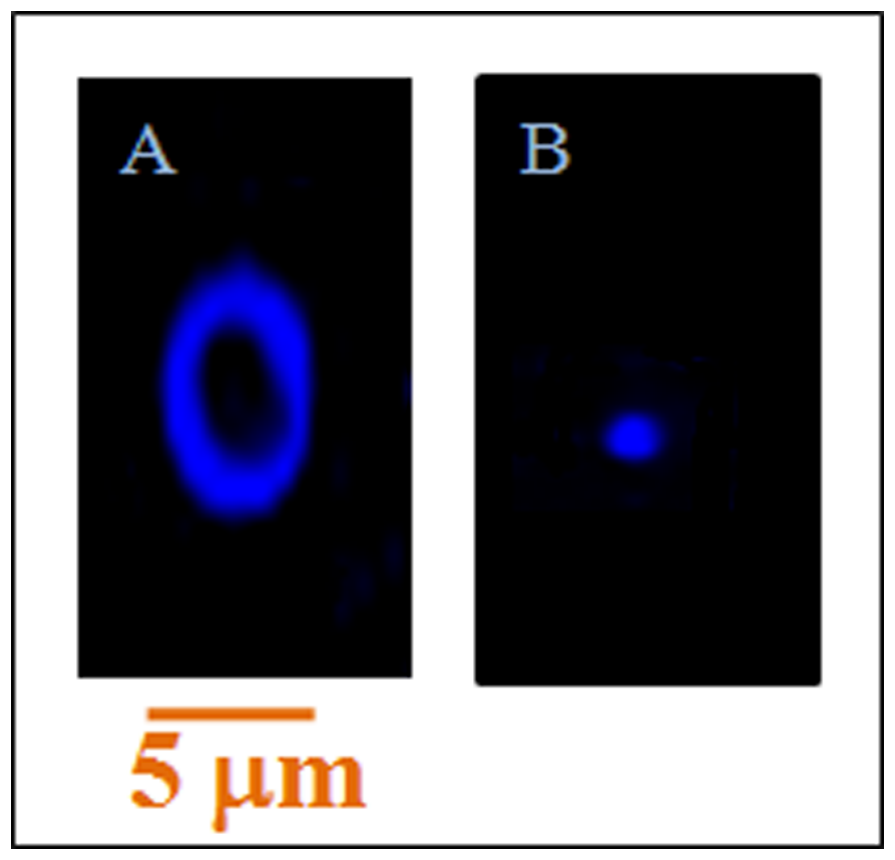
Fluorescence microscopic images. Fluorescence microscopy images of DNA (0.5 μM) labeled DAPI (0.5 μM) in (A) absence and (B) presence of Gua-IL (1 mM).

In order to visualize this DNA compaction process, we have executed the fluorescence microscopy study of dye (DAPI) labeled DNA in presence and absence of Gua-IL. Here it is pertinent to mention that getting microscopic image of ct-DNA is difficult, because the resolution of fluorescence microscope is limited to few micro-meters. Therefore, we have selected plasmid DNA (4600 Kbp) as a model DNA for the visualization of this compaction process. DAPI labeled DNA exhibits a circular morphology (Figure 8), which is quite evident for a plasmid DNA. However, when Gua-IL is added to the dye labeled DNA solution, a distinctive change in DNA volume is observed (Figure 8).

## Discussion

Reduction in fluorescence intensity of EB in presence of Gua-IL suggests that intercalated EB molecules are coming out from the DNA (Figure 2A). Apparently, it can be thought that Gua-IL may intercalate in between the base pairs and replaces EB molecules. Here it is pertinent to mention that previously Cheng *et al.*^36^ reported the intercalation of [Bmim][Cl] ionic liquid in between DNA base pairs. In [Bmim][Cl] ionic liquid, [Bmim] moiety consists of imidazolium ring with delocalized π-clouds, Which can participate in stacking interaction with nucelobases. Notably, intercalators have features like labile, planar π-electrons, which can stack efficiently with nucelobases^49^,^50^. Being a triangular non-aromatic structure, guanidinium cation cannot act as an intercalator. It is noteworthy to mention that the effect of Gua-IL comes into the picture from milli-molar concentration range in both the cases (EB and DAPI). A normal intercalation or minor groove binding process is supposed to be effective from very low concentration (even in micro-molar concentration range) range, which is not observed here (decrement in emission maximum for EB and/or DAPI is taking place from a certain concentration (>1 mM)). Hence, the observed results create a doubt about the possibility of the intercalation and/or minor groove binding mode of Gua-IL with DNA. Notably, the possibility of some molecular aggregation of Gua-IL can be speculated from the DLS, FE-SEM and TEM studies at higher concentration (≥1 mM) of Gua-IL, which might lead to the displacement of dye from their respective positions in DNA. Here it is relevant to mention that we have also noticed identical observation with shorter DNA (DD, 5′-d-(CGCGAATTCGCG)_2_-3′) suggesting the similar impact of Gua-IL on both short (Figure S3) and long DNA.

Like steady-state fluorescence results, anisotropy results also show identical observation in milli-molar concentration range. But, the changes in anisotropy are not so prominent like steady-state intensity profiles. This is possibly due to the enhanced viscosity of the respective dye-DNA solution in presence of increasing Gua-IL concentration. In case of DAPI-DNA, anisotropy decrement in presence of Gua-IL is less and it is attributed to the lesser extent of DAPI displacement, which is corroborative with fluorescence displacement assay results. Decreasing trend in anisotropy values for both of the dyes (EB and DAPI) implies the similar possibilities like either replacement of the dyes due to binding of Gua-IL at the respective positions or some molecular aggregation of Gua-IL, which displaces the dyes from DNA.

In addition to steady-state fluorescence measurements, we have also performed circular dichroism study, which provides information about the structural changes of DNA in presence of Gua-IL. In case of DNA-EB system, no evident alteration in peaks is observed at lower concentration (up to 50 μM) of Gua-IL (Figure 4A). Afterwards, we have witnessed some evident changes in the spectra, and interestingly, >1 mM Gua-IL concentration, induced signal (at 303 nm) totally disappeared. Moreover, above this concentration range (>1 mM), we have also observed the CD signal decrement (Figure 4B) for both of base stacking and helicity bands, which are not the characteristic features of any intercalator in DNA. Thus, these observations again reinforce our claim that Gua-IL is not intercalating between the base pairs of DNA. However, the above CD results suggest that structural reformation of ct-DNA takes place by Gua-IL. Analogous experiments with DNA-DAPI also demonstrate similar results (Figure 4C), where any kind of alteration was only perceived in milli-molar concentration (>1 mM) range. Another interesting fact is that induced signal at 360 nm did not vanish at maximum addition of Gua-IL inferring that Gua-IL cannot replace all DAPI molecules from the minor groove of ct-DNA. These results are well corroborative with the steady state results, where we have also observed Gua-IL was unable to replace all of DAPI molecules from DNA. In a nutshell, CD results confirmed that Gua-IL has some structural influence on DNA after a certain concentration (1 mM) except intercalation and minor groove binding modes of interaction.

Dynamic light scattering study of ct-DNA (Figure 5A) exhibits an unimodal peak at 350 nm, which attributes to the translational mode of extended DNA^37^. With the milli-molar addition of Gua-IL (>1 mM) to the system, the intensity weighted distribution peak (Figure 5A) appears to be bimodal with hydrodynamic peaks at ~60 nm and ~250 nm. From DLS study of Gua-IL alone (in absence of DNA), it is evident that self aggregation of Gua-IL takes place above 1 mM concentration (i.e. CAC), and the size of the aggregated structure is around ~10 nm at 1 mM concentration. Moreover, the size of the aggregated structure increases as the concentration of Gua-IL increases, and the size of the aggregated structure is ~35 nm at 40 mM concentration of Gua-IL. Notably, opposite finding is observed when Gua-IL is added to the DNA system. Therefore, the two different size distributions appeared at ~60 nm and ~250 nm in DNA-IL system cannot be due to the aggregation process of Gua-IL itself. Notably, DNA compaction by surfactants, dendrimers also perceived similar size distribution^47^,^51^. Thus, the appearance of peak at lower hydrodynamic radius (i.e. ~60 nm) refers to some anomalous structure of DNA, which might represent the compacted or condensed state of DNA by Gua-IL. The small peak at ~60 nm gradually shifts to lower hydrodynamic radius with increase in concentration of Gua-IL, which accounts for the more compacted structure of DNA. The extended peak for DNA (at 350) nm in DNA-IL system also shifts towards lower hydrodynamic radius with increasing Gua-IL in solution. This reduction in hydrodynamic radius may be due to the spontaneous binding of guanidinium cation with phosphate group of DNA^37^. To explore the aggregation mechanism of Gua-IL, we have executed the similar DLS study with Gua HCl, which has similar cationic part (i.e. Guanidium ion) but different anionic part. Interestingly, any intensity weighted peak at similar concentration range was not present for Gua HCl, which reflects the involvement of anionic part of Gua-IL in micellar kind of aggregation, instead of guanidinium cationic part. Thus, we believe that anionic part of Gua-IL, which contains hydrophobic -CF_2_CF_3_ group, may aggregate like micelles in order to avoid unfavorable interactions with aqueous medium. This type of IL aggregation is a well known feature in literature^39^ and the zeta potential study also reflects the same. Zeta potential result is also well corroborative with our previous DLS, and steady-state results. It shows an inflection point near 1 mM Gua-IL concentration (Figure 5B) and afterwards zeta potential value again increases with increasing Gua-IL concentration indicating the formation of higher order compacted structure from those globular structure. The shifting of DNA melting temperature (Figure 6) at lower site (from 41°C to 21°C) in presence of Gua-IL refers to the decrement in stability of these compacted DNA due to loss in double helical conformation, which is also reflected in CD studies. Interestingly, this trend in melting studies is comparable to the compaction process mediated by nano-particles^12^. In basis of all our experimental observations, we infer that the compaction is taking place by the electrostatic dragging of DNA strands (by the guanidinium cation) to the aggregated surface of Gua-IL, which is formed by the hydrophobic chains of the Gua-IL anionic part. Accumulating steady state fluorescence, DLS, CD and melting results, we anticipate that Gua-IL does not involve in intercalation and/or minor groove binding, rather induces DNA structure in such a way that it leads to compaction from coil to globule state.

The FE-SEM studies provide direct pictorial evidence of this compaction process (Figure 7). A super coiled morphology appears for ct-DNA, which is quite obvious for a long DNA like calf thymus (Figure 7A). A obvious alteration in the morphology is observed when Gua-IL is introduced to the system at a molar ratio of 20 ([Gua-IL]/[DNA]), indicating the initiation process for DNA compaction (Figure 7B). At a higher molar ratio ([Gua-IL]/[DNA]) of 40, a globular morphology is observed (Figure 7C). This morphology compliments well with the earlier reported DNA compaction morphology^52^, and the diameter of these globules are in good agreement with the diameter (around 40 nm) obtained from DLS study. Both the SEM and TEM studies confirm the formation of aggregated Gua-IL structure having spherical shaped morphology. Moreover, there exists a distribution of aggregates size having smallest diameter of 30 nm. This size is very much comparable with that of DLS study and the morphology is also quite similar to the micelles^53^. Notably, due to complete drying of Gua-IL sample for SEM/TEM studies, there is a high chance for the formation of larger aggregates (around 180 nm) from the smaller aggregates/micelles (around 30 nm), which is not observed in DLS study. The difference in size distribution (for Gua-IL) observed between DLS and microscopic images are due to the different states of samples. DLS was carried out in solution phase, whereas SEM study has been studied in dried condition where aggregations in between micelles are quite obvious.

Fluorescence microscopy images (Figure 8) render a clear evidence for this compaction process induced by Gua-IL. DAPI labeled DNA shows a circular structure, which is evident for a plasmid DNA (Figure 8). Interestingly, this circular structure significantly gets compacted by the Gua-IL (Figure 8). In a nutshell, FE-SEM and fluorescence microscopy images have provided direct evidence for the Gua-IL induced DNA compaction process.

### Plausible compaction mechanism

**Figure 9:**
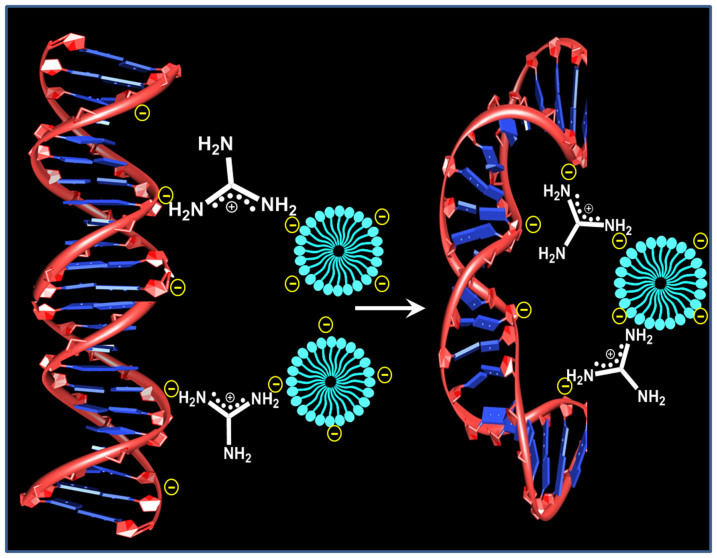
DNA compaction scheme. A plausible DNA compaction mechanism.

In literature, there are reports about different interaction modes of IL with DNA. *Cheng et al.* shows the intercalation binding mode of [Bmim][PF_6_] with DNA^36^. An aggregation induced DNA perturbation by [Bmim][Cl] IL was reported by Ding *et al*^37^. Later on in 2012, a groove binding mechanism of RTIL in DNA was proposed by Chandran *et al.*^38^ They reported that [Bmim] cation interacts with minor groove of DNA through various non covalent interactions. Herein, for the first time we have perceived the excellent DNA compaction property of Gua-IL. The plausible compaction mechanism has been proposed based on our results obtained from fluorescence, CD, DLS, FE-SEM, TEM and confocal fluorescence microscopy experiments. Being a negatively charged stiff polymer, compaction of DNA needs the neutralization of its negatively charged phosphate group, and for this neutralization process counter ion valency (Z) of compacting agent should be greater or equal to 3 (Z ≥ 3) in accordance with Manning–Oosawa condensation theory^54^,^55^. In our case, Gua-IL possesses mono-valency in micro-molar, but in milli-molar concentration range it acts as multivalent ion (Z ≥ 3) due to the formation of micellar type aggregates. All the other experimental methods employed here (fluorescence displacement assay experiments, circular dichroism) also established the fact that above a certain concentration (>1 mM) of Gua-IL all the spectral changes take place. Interestingly, we have noticed that 1 mM is the CAC of Gua-IL from DLS study. Therefore, we strongly believe that micellar like aggregates of Gua-IL is taking major role for the compaction process of ct-DNA. Notably, guanidium cations have two positively charged centres due to its intramolecular resonance. One positively charged part neutralizes the negatively charged phosphate (PO_4_^3−^) group of DNA and another part involves in electrostatic interaction with its counter anion. When these anions form a micellar kind of aggregate after CAC, then the counter positively charged ions situated at micellar surface drag the negatively charged DNA strands to the micellar surface due to electrostatic attraction between opposite ion pairs (Figure 9). This process leads to wrapping of several parts of DNA around these micellar kinds of aggregates. With the increase in Gua-IL concentration, the number of loops increases and finally results the DNA globular structure by collapsing the intra/interstrand repulsion between DNA strands and instigates the structural transition from coil to globule state.

It is also well known fact that ionic liquids can generate certain extent of osmotic stress in solution, which is achieved due to the reduction in the dielectric permittivity of the solution^26^,^56^. Here it is pertinent to mention that Mel'nikov *et al.* suggested an enhancement in electrostatic attraction between DNA and counter ion due to the lowering of dielectric permittivity^26^. Similarly, here the process of electrostatic interactions between negatively charged phosphate groups of DNA and polyvalent ion (due to the formation of micellar aggregation by the anionic counter part of Gua-IL) become even more facile due to the diminution in dielectric permittivity of the solution, and thus attraction between DNA strands increases. Therefore, we believe that in addition to the polyvalent neutralization by micellar arrangements (described previously); this osmotic stress might also be responsible for this compaction phenomenon. The proposed compaction mechanisms may comparable to the DNA compaction by histone protein, nanoparticles, dendrimers and PEG^13^.

## Conclusion

Current work deals with two important findings of interaction between DNA and green solvent, RTIL. Firstly, the mode of interaction between DNA and IL and secondly, an excellent DNA compacting ability of RTIL have been revealed. To the best of our knowledge, it is the first ever report of DNA compaction mediated by RTIL, namely, guanidinium tris(pentafluoroethyl)trifluorophosphate (Gua-IL).

Dye displacement study through fluorescence and CD spectroscopy imply that Gua-IL has reasonable impact on both intercalation position and minor groove of ct-DNA, which is a characteristic feature of DNA compaction. Further, DLS study manifests the specific role of anionic part of Gua-IL in compaction process, and thereby helps us to propose plausible mechanism of compaction process. UV melting study shows a decrement in melting temperature of DNA with increasing extent of compaction. Interestingly, we could probe the compaction process through field emission scanning electron microscopic (FE-SEM) and confocal fluorescence microscopic studies, which validate and provide a direct proof of this compaction process instigated by Gua-IL. Based on all the spectroscopic and microspcopic evidences, we propose that the counter positively charged guanidium cations situated at the surface of micellar like aggregates (formed by the anionic part of Gua-IL after CAC) drags the negatively charged DNA strands to the micellar surface due to the electrostatic attraction as well as diminution in dielectric permittivity of the solution. Eventually, the above mentioned effect reduces the intrastrand and/or interstrand DNA repulsion and triggers the structural transition from coil to globular state.

The important essence of this work is that a new class of DNA compaction agent i.e. room temperature ionic liquid (RTIL) has been identified and is found to be very effective both for shorter (dodecamer DNA), larger (ct-DNA) DNA and plasmid DNA.

## Methods

Ethidium bromide (EB), 4′,6-diamidino-2-phenylindole (DAPI), sodium salt of calf thymus DNA (ct-DNA) were purchased from Sigma Aldrich and used without any purification. Dickerson dodecamer (DD, 5′-d-(CGCGAATTCGCG)_2_-3′) was purchased from integrated DNA technology (IDT) and used without any purification. The purity of ct-DNA was checked by taking ratio of the absorbance at 260 nm to that at 280 nm, which was found to be 1.80. This indicates absence of any kind protein in ct-DNA. Plasmid DNA (4600 Kbp), which was extracted from E. coli DH5α bacteria, was purchased from geneOmbio Technologies, India. The ionic liquid, guanidinium tris(pentafluoroethyl)trifluorophosphate was purchased from Merck, Germany. All the buffers and samples were prepared using millipore water. Concentrations of EB and DAPI were determined using molar extinction coefficients of 5,450 M^−1^ cm^−1^ at 480 nm^57^ and 27,000 M^−1^ cm^−1^ at 342 nm^58^, respectively. Concentration of ct-DNA and plasmid DNA were measured using the molar extinction coefficient 6600 M^−1^ cm^−1^ at 260 nm per base pair. DNA was annealed by heating DNA samples at 90°C for about 5 minutes and then gradually cooled down to room temperature. All the ct-DNA samples were prepared either using 5 mM phosphate buffer (pH = 7.4) or 5 mM Tris-HCl buffer (pH = 7.4).

Buffer pH was determined by silicon micro sensor pocket sized pH meter (ISFETCOM. Co. Ltd., Japan). Steady state fluorescence and anisotropy measurements were carried out in FluoroMax-4 spectrofluorimeter (Horiba Scientific, U.S.A.). All the steady state studies were carried out by adding DNA in respective dyes and it is followed by Gua-IL addition. Circular dichroism (CD) spectra were recorded on a J-815 CD (JASCO, U.S.A.) instrument at 25°C. The data were collected at 1 nm intervals with 1 nm band width. All the measurements were taken in 0.2 cm path length cuvette with 400 μL sample volume. Each CD profile is an average of 3 scans of the same sample collected at a scan speed 100 nm/min, with a proper baseline correction from the blank buffer. CD studies were monitored by the addition of respective dyes in ct-DNA which is followed by Gua-IL addition. All the size determination and zeta potential experiments were carried out by dynamic light scattering instrument, using a Nano ZS-90 apparatus utilizing a 633 nm red laser (at 90° angle) from Malvern instruments. For size and zeta potential determination, we have used polystyrene cuvette (from sigma) and disposable capillary cell (DTS1070), respectively. Thermal melting study was executed using Varian Cary 300 Bio UV-Vis Spectrophotometer (Thermo Fisher Scientific, U.S.A.). Thermal melting was monitored at 290 nm with heating rate 1°C/min. Here, we used very small concentration of DNA (~1 μM) due to avoid the absorbance saturation. The melting temperature (Tm) was determined from the sigmoidal curve fit of the melting profile. The field emission scanning electron microscope (FE-SEM) images of DNA and DNA-Gua-IL systems were taken from a ZEISS, Ultra Plus, where all the samples were prepared by drop casting the solution in silicon wafers. High resolution transmission electron microscope (HR-TEM) images were collected with a Technai-300 transmission electron microscope opened at an accelerating voltage of 200 kV. The samples for TEM images were drop casted on a Cu grid and left for drying for 12 hours. Fluorescence microscopy images were obtained from Carl Zeiss Axio Observer.A1 equipped with an oil immersion lens and analyzed using ZEN 2014 software. Plasmid DNA (0.5 μM) and DAPI (0.5 μM) were mixed in 10 mM Tris HCl buffer (pH 7.6). Then Gua-IL (500 μM) added in the above mentioned solution. Samples were kept for 20-30 min prior to imaging. All the glass microscope slides and coverslips were immersed into ethanol for overnight before the experiment. All the experimental measurements were performed at ~25°C.

## Supplementary Material

Supplementary Information A Green Solvent Induced DNA Package

## Figures and Tables

**Figure 1:**
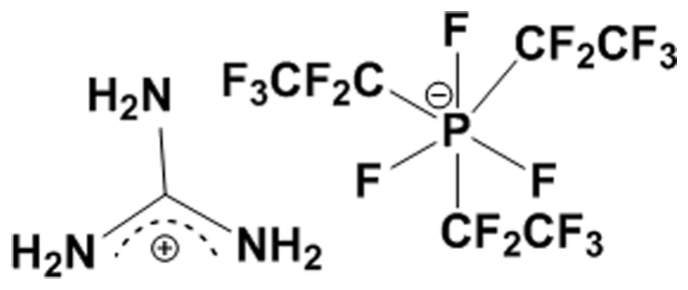
Structure of Gua-IL. Chemical structure of guanidinium tris(pentafluoroethyl)trifluorophosphate (Gua-IL).
