# Supplementary material for: A Green Solvent Induced DNA Package
Source: Sci Rep. 2015 Mar 16;5:9137. doi: 10.1038/srep09137 (PMC5378943; doi:10.1038/srep09137)
Supplement: Supplementary Information — A Green Solvent Induced DNA Package [file srep09137-s1.doc]

Supporting Information

**A Green Solvent Induced DNA Package**

Sagar Satpathi, Abhigyan Sengupta, Hridya V. M., Krishna Gavvala, Raj Kumar Koninti, Bibhisan Roy, Partha Hazra^*^

Department of Chemistry, Indian Institute of Science Education and Research (IISER)-Pune,

Pune (411008), Maharashtra,

India

*Corresponding author E-mail: [*p.hazra@iiserpune.ac.in*](mailto:p.hazra@iiserpune.ac.in)

Tel.: +91-20-2590-8077; Fax: +91-20-2589 9790

**Figure S1.** Fluorescence spectra of dyes with increasing concentration of ct-DNA (A) EB (B) DAPI.

**Figure S2.** Fluorescence spectra of dyes with increasing concentration of Gua-IL (A) EB (B) DAPI.

**Figure S3.** Fluorescence spectra of DD-DNA bound dyes with increasing concentration of Gua-IL (A) EB (B) DAPI.

**Figure S4.** Circular dichroism spectra of ct-DNA with gradual addition of EB.

**Figure S5.** Circular dichroism spectra of DD-DNA bound dye with gradual addition of Gua-IL (A) EB (B) DAPI.

**Figure S6.** Intensity weighted distribution peak (measured by DLS) with increasing concentration of Gua-IL.


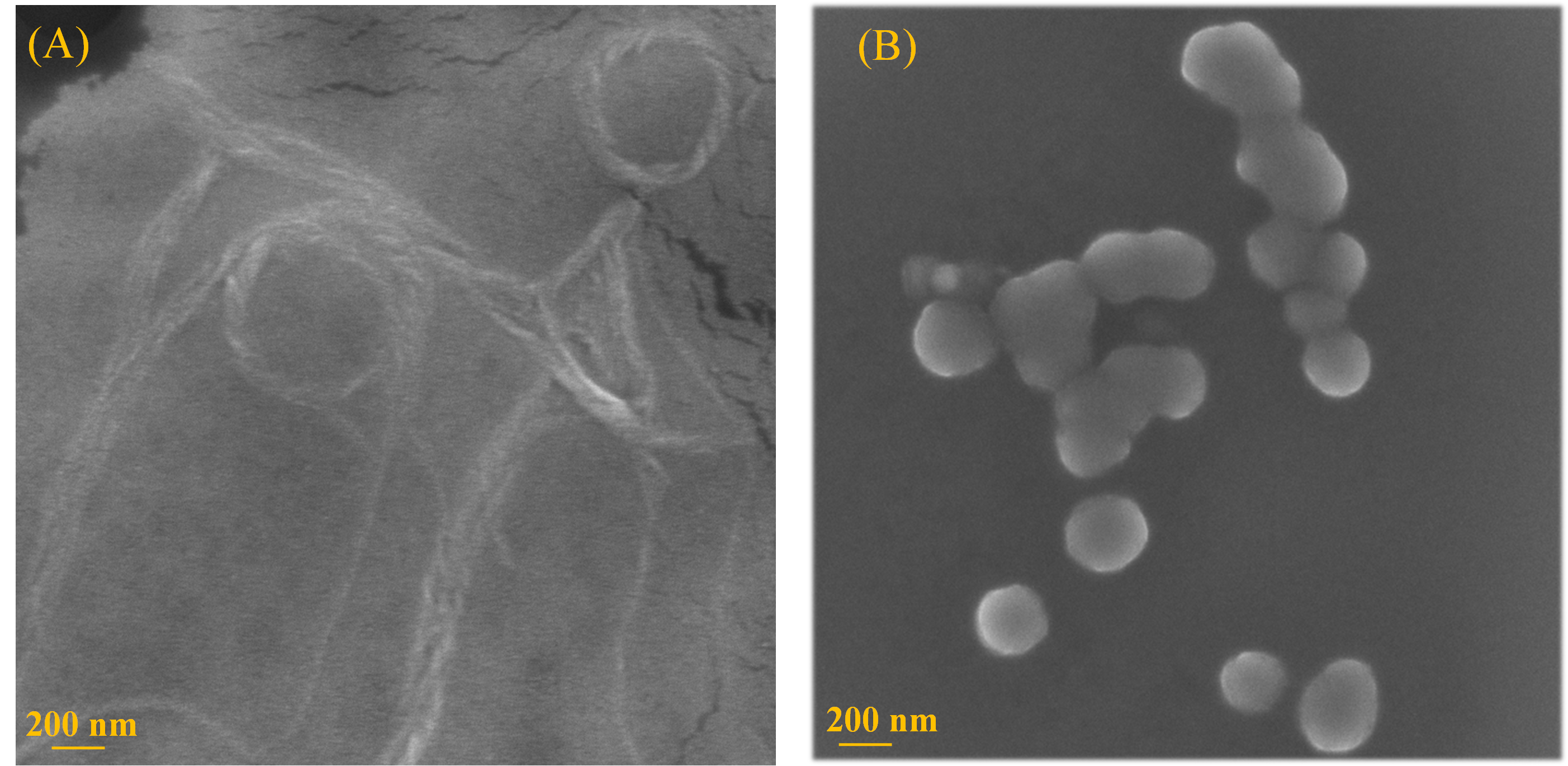


**Figure S7.** FE-SEM images of (A) ct-DNA (B) Gua-IL.
